# Supplementary material for: PPARɣ drives IL-33-dependent ILC2 pro-tumoral functions
Source: Nat Commun. 2021 May 5;12:2538. doi: 10.1038/s41467-021-22764-2 (PMC8100153; doi:10.1038/s41467-021-22764-2)
Supplement: Supplementary file 1 — Supplementary Information [file 41467_2021_22764_MOESM1_ESM.pdf]

Supplementary Information

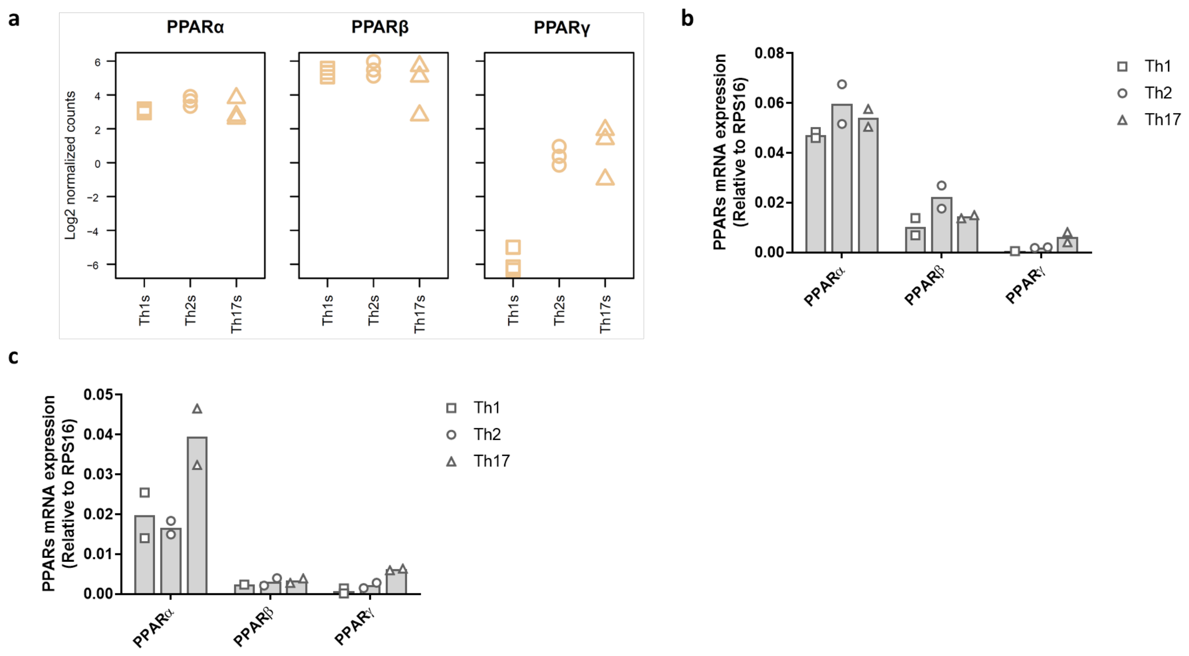

Supplementary Figure 1: Expression of PPARs in human CD4 Th subsets. **a, b,** Expression of PPARs in freshly sorted CD4 Th subsets assessed by RNA-seq (**a**) (n = 3), and qPCR (**b**) analysis (n=2). **c,** Expression of PPARs in *in vitro* expanded Th subsets assessed by qPCR analysis (□ Th1, ○ Th2, Δ Th17) (n = 2). Each symbol represents one individual donor. Source data are provided as a Source Data file.

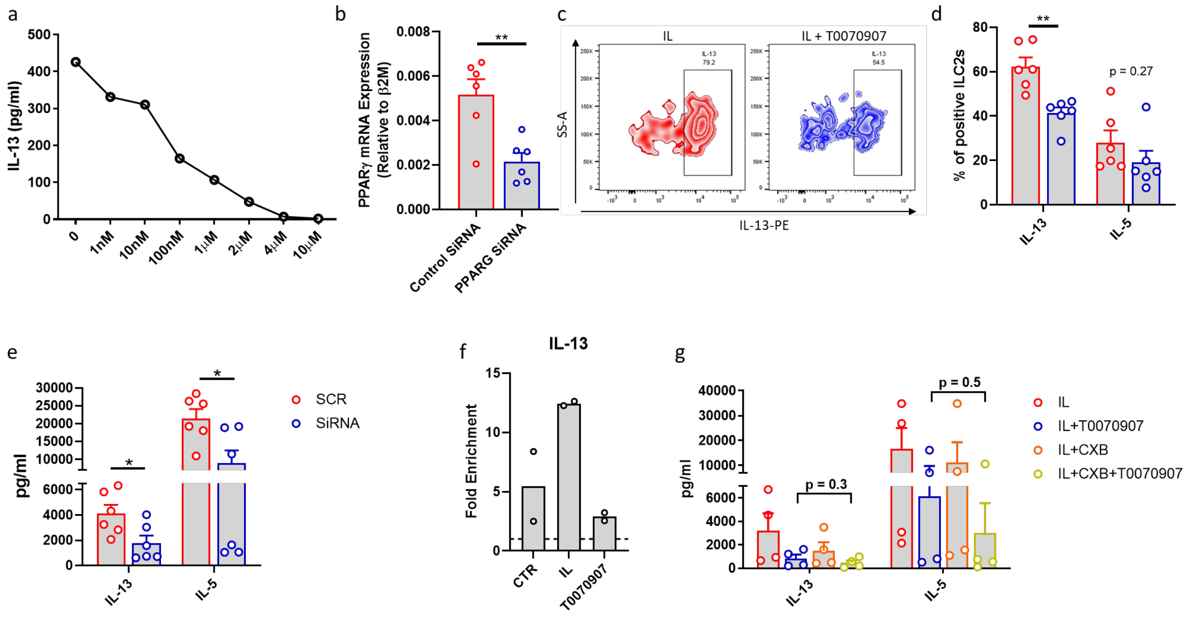

Supplementary Figure 2: Effect of T0070907 on ILC2-derived IL-13 secretion, PPARγ silencing in ILC2s and synergistic effect with Celecoxib. **a,** Quantification of IL-13 assessed by Legendplex analysis in cell culture supernatants of ILC2s upon treatment with increasing concentrations of T0070907. **b,** Silencing of PPARγ following transfection qPCR analysis of ILC2s transfected with scramble siRNA (○ SCR) or PPARγ SiRNA (○) (n = 6; **P= 0.0027). **c,** Representative example of flow cytometry analysis of ILC2-derived IL-13 upon transfection with SCR and PPARγ SiRNA. **d**, Frequencies of IL-13 and IL-5 positive cells among ILC2s, after silencing with SCR (○) and PPARγ SiRNA (○) (n = 6; **P= 0.0074). **e**, Quantification of IL-13 and IL-5 assessed by Legendplex analysis in cell culture supernatants of ILC2s upon silencing with SCR (○) and PPARγ SiRNA (○) (n = 6; IL-13 *P= 0.0459, IL-5 *P= 0.0187). **f,** ChIP-qPCR analysis of PPARγ occupancy on IL-13 promoter in untreated ILC2s (CTR) and in ILC2s treated with the cytokine cocktail (IL) and T0070907 (n = 2). **g,** Quantification of IL-13 and IL-5 by Legendplex in cell culture supernatants of ILC2s untreated (○) and upon treatment with T0070907 (○) and Celecoxib (CXB) (○) and their combination (○) (n = 4). Each symbol represents one individual donor. Data are shown as mean ± SEM and were analyzed by Wilcoxon (**b**) or two-way (**d, e, g**) ANOVA tests. Source data are provided as a Source Data file.

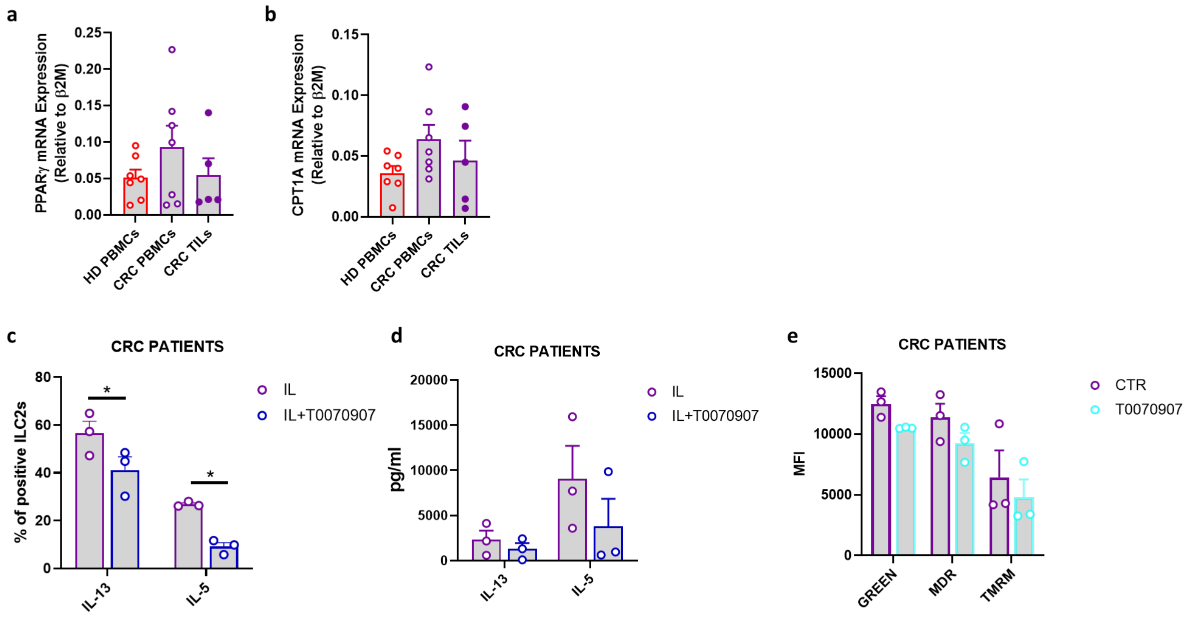

Supplementary Figure 3: Expression of PPARγ in freshly sorted ILC2 and effect of T0070907 in *in vitro* expanded ILC2s from CRC patients. **a,b,** Expression of PPARγ and CPT1A in freshly sorted ILC2s from HD PBMCs (○), CRC patients' PBMCs (○) or TILs (●) assessed by qPCR analysis (HDs and CRC PBMCs n = 7, CRC TILs n = 5). **c,** Frequencies of IL-13 and IL-5 positive cells among ILC2s, after cytokine stimulation (IL) and treatment (○) or not (○) with T0070907 (n = 3; IL-13 *P= 0.0468, IL-5 *P= 0.0242). **d**, Quantification of IL-13 and IL-5 assessed by Legendplex in cell culture supernatants of ILC2s upon cytokine stimulation (IL) and treatment (○) or not (○) with T0070907 (n = 3). **e,** Quantification of MitoTracker Green, MitoTracker Deep-red and TMRM in control ILC2s (CTR ○) and in ILC2s after T0070907 treatment (○) for 48 hours (n = 3). Each symbol represents one individual. Data are shown as mean ± SEM and were analysed by one- (**a, b**) or two-way (**c-e**) ANOVA tests. Source data are provided as a Source Data file.

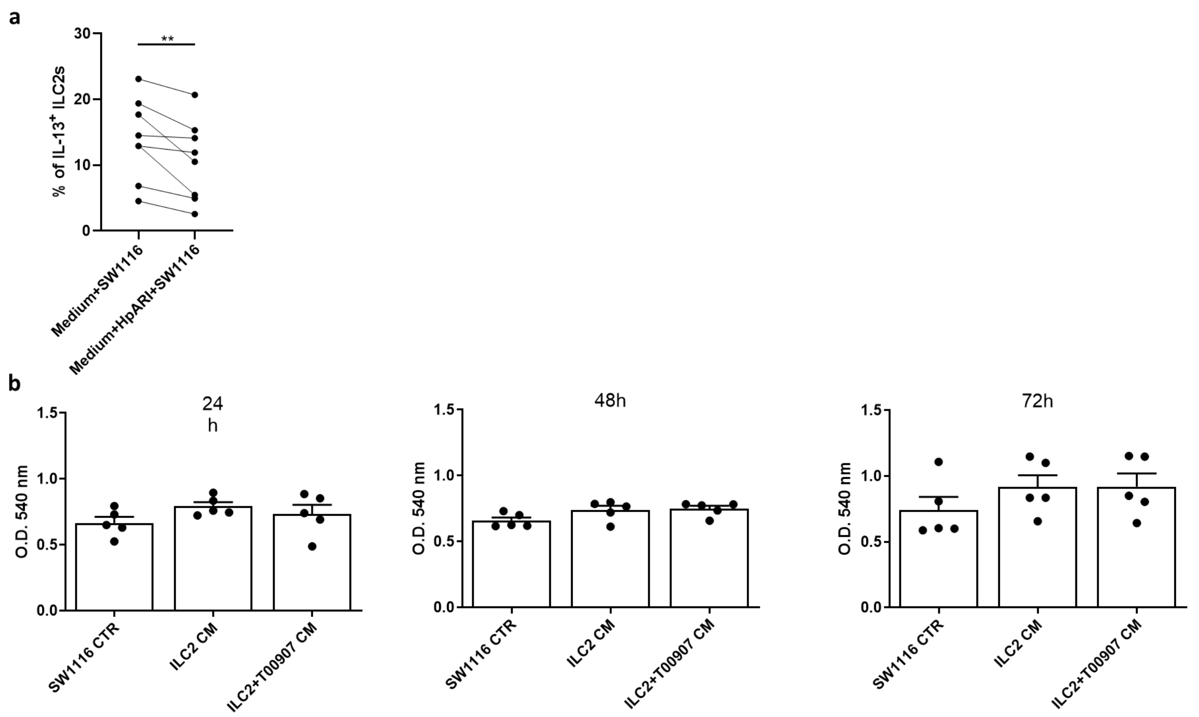

Supplementary Figure 4: Effect of HpARI in ILC2s co-cultured with SW1116 CRC cells and effect of ILC2 CM on SW1116 proliferation rate. **a,** Frequencies of IL-13 positive ILC2s upon co-culture with SW1116 CRC cells and addition of HpARI (n = 8; **P= 0.0078). **b,** Proliferation rate was assessed by MTT assay in SW1116 CRC cells after incubation with ILC2s or ILC2+T0070907 CM (n = 5). Each symbol represents one individual donor or sample. Data are shown as mean ± SEM and were analyzed by Wilcoxon (**a**), one-way (**b**) ANOVA tests. Source data are provided as a Source Data file.

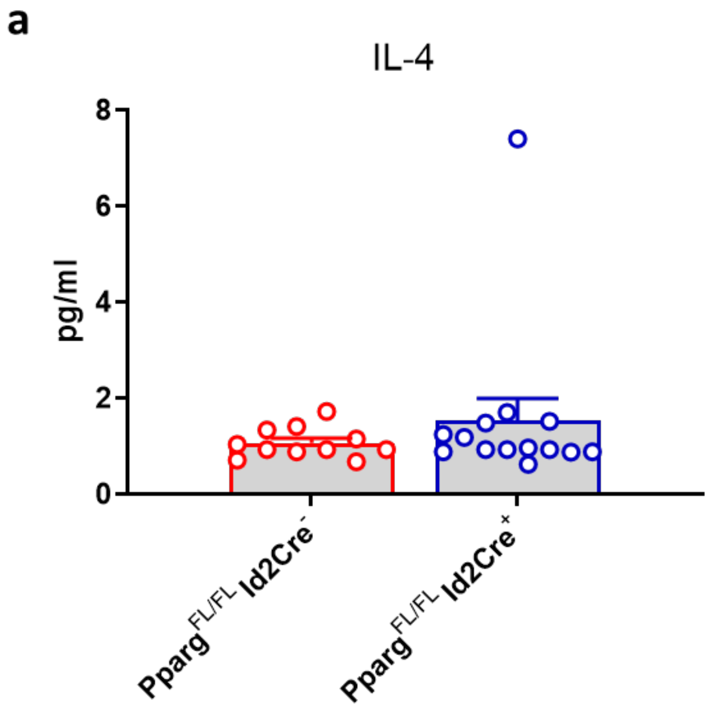

Supplementary Figure 5: IL-4 secretion in ILC2s sorted from Pparg$^{fl/fl}$ Id2Cre mice. **a,** Quantification of IL-4 assessed by Legendplex analysis in cell culture supernatants of ILC2s sorted from the lungs of Pparg$^{fl/fl}$ Id2Cre mice (Pparg$^{fl/fl}$Id2CreER^{T2} positive (○) or CreER^{T2} negative mice (○)) (Pparg$^{fl/fl}$Id2CreER^{T2} negative n = 11, Pparg$^{fl/fl}$Id2CreER^{T2} positive n = 14). Each symbol represents one individual donor. Data are shown as mean ± SEM and were analysed by Wilcoxon tests. Source data are provided as a Source Data file.

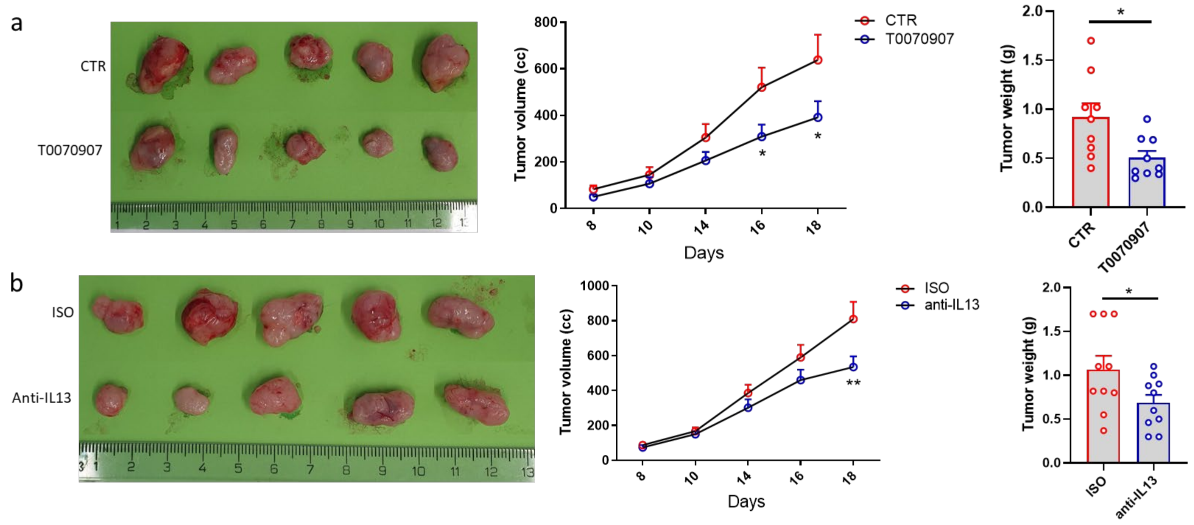

Supplementary Figure 6: Effects of T0070907 and anti-IL-13 neutralizing antibody in CRC progression. **a,** Representative image of tumor size (left), tumor development expressed as tumor volume (middle) and weight (right) in control (○) and T0070907 (○) treated tumor-bearing mice (n=9; Tumor Volume 16 days *P= 0.0462, 18 days *P= 0.0132, Tumor Weight *P= 0.0117). **b,** Representative image of tumor size (left), tumor development expressed as tumor volume (middle) and weight (right) in isotype (○) and anti-IL13 neutralizing antibody (○) treated tumor-bearing mice (n=10; **P= 0.0018, *P= 0.0480). Each symbol represents one individual mouse. Data are shown as mean ± SEM and were analysed by Wilcoxon (**a right, b right**) or two-way (**a middle, b middle**) ANOVA tests. Source data are provided as a Source Data file.

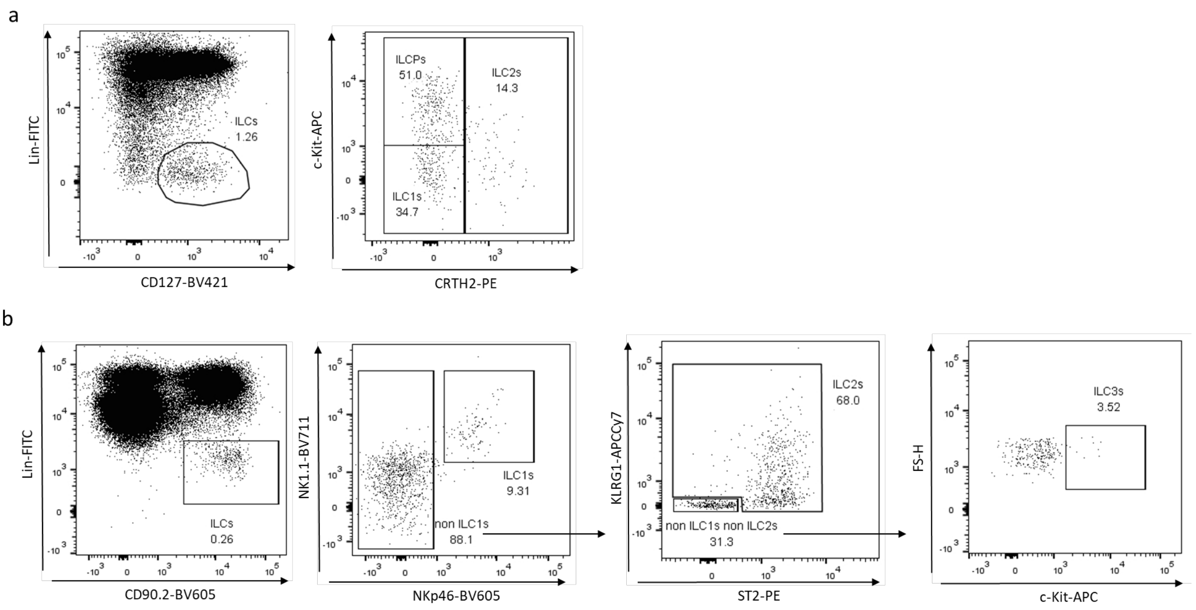

Supplementary Figure 7: Gating strategy used for ILC identification by Flow Cytometry. **a,** Human ILCs were identified as lineage negative $CD127^+$ cells; within this population, we identified ILC1s as $c\text{-}Kit^-$ $CRTH2^-$, ILC2s as $CRTH2^+$ $c\text{-}Kit^{+/-}$, ILCPs as $c\text{-}Kit^+$ $CRTH2^-$ cells for PPARγ expression presented on Fig. 1b-d. **b,** Murine ILCs were identified as $CD45^+$, Lin^-, $CD90.2^+$. ILC subsets are identified based on the expression of NK.1.1 and NKp46 surface markers: ILC1s are identified as double positive population. By gating on the double negative population, we identified ILC2s according to the expression of ST2 and KLRG1 and ILC3s according to the expression of c-Kit for PPARγ expression presented on Fig 6a. The same strategies were used for the *in vitro* and *in vivo* experiments with expanded ILC2s.
