# Supplementary material for: PPARɣ drives IL-33-dependent ILC2 pro-tumoral functions
Source: Nat Commun. 2021 May 5;12:2538. doi: 10.1038/s41467-021-22764-2 (PMC8100153; doi:10.1038/s41467-021-22764-2)
Supplement: Supplementary file 3 — Descriptions of Additional Supplementary Files [file 41467_2021_22764_MOESM3_ESM.pdf]

Descriptions of Additional Supplementary Files

Supplementary Data 1
Description: Human Peroxisome proliferator response element (PPRE) motif search in gene promoters.

Supplementary Data 2
Description: Mouse Peroxisome proliferator response element (PPRE) motif search in gene promoters.
