# Supplementary material for: PPARɣ drives IL-33-dependent ILC2 pro-tumoral functions
Source: Nat Commun. 2021 May 5;12:2538. doi: 10.1038/s41467-021-22764-2 (PMC8100153; doi:10.1038/s41467-021-22764-2)
Supplement: Supplementary file 6 — Reporting Summary [file 41467_2021_22764_MOESM6_ESM.pdf]

nature research

Corresponding author(s): Camilla Jandus

Last updated by author(s): Mar 11, 2021

Reporting Summary

Nature Research wishes to improve the reproducibility of the work that we publish. This form provides structure for consistency and transparency in reporting. For further information on Nature Research policies, see our Editorial Policies and the Editorial Policy Checklist.

Statistics

For all statistical analyses, confirm that the following items are present in the figure legend, table legend, main text, or Methods section.

n/a	Confirmed	
☐	☒	The exact sample size (n) for each experimental group/condition, given as a discrete number and unit of measurement
☒	☐	A statement on whether measurements were taken from distinct samples or whether the same sample was measured repeatedly
☐	☒	The statistical test(s) used AND whether they are one- or two-sided<br>*Only common tests should be described solely by name; describe more complex techniques in the Methods section.*
☒	☐	A description of all covariates tested
☒	☐	A description of any assumptions or corrections, such as tests of normality and adjustment for multiple comparisons
☐	☒	A full description of the statistical parameters including central tendency (e.g. means) or other basic estimates (e.g. regression coefficient) AND variation (e.g. standard deviation) or associated estimates of uncertainty (e.g. confidence intervals)
☐	☒	For null hypothesis testing, the test statistic (e.g. F, t, r) with confidence intervals, effect sizes, degrees of freedom and P value noted<br>*Give P values as exact values whenever suitable.*
☒	☐	For Bayesian analysis, information on the choice of priors and Markov chain Monte Carlo settings
☒	☐	For hierarchical and complex designs, identification of the appropriate level for tests and full reporting of outcomes
☒	☐	Estimates of effect sizes (e.g. Cohen's d, Pearson's r), indicating how they were calculated

Our web collection on statistics for biologists contains articles on many of the points above.

Software and code

Policy information about availability of computer code

Data collection	No software was used
Data analysis	GraphPad Prism Version 6<br>FlowJo software (TreeStar V.10)<br>EMMENU software Version 1.2<br>Legendplex software (version 8.0)<br>HOMER software (v.4.11,)

For manuscripts utilizing custom algorithms or software that are central to the research but not yet described in published literature, software must be made available to editors and reviewers. We strongly encourage code deposition in a community repository (e.g. GitHub). See the Nature Research guidelines for submitting code & software for further information.

Data

Policy information about availability of data

All manuscripts must include a data availability statement. This statement should provide the following information, where applicable:

- Accession codes, unique identifiers, or web links for publicly available datasets
- A list of figures that have associated raw data
- A description of any restrictions on data availability

mRNA sequencing data deposited in the ArrayExpress under accession number E-MTAB-8494
STRING database (https://string-db.org/)
Eukaryotic Promoter Database (human genome version Dec 2013 GRCh38, mouse genome version Mar 2012 mm10; https://epd.epfl.ch//index.php)
Jaspar2020 database (matrix IDs: MA0065.1 (human), MA0065.2 (mouse), http://jaspar.genereg.net/)

Field-specific reporting

Please select the one below that is the best fit for your research. If you are not sure, read the appropriate sections before making your selection.

- [x] Life sciences
- [ ] Behavioural & social sciences
- [ ] Ecological, evolutionary & environmental sciences

For a reference copy of the document with all sections, see nature.com/documents/nr-reporting-summary-flat.pdf

Life sciences study design

All studies must disclose on these points even when the disclosure is negative.

Sample size	Sample size was estimated with G power software in order to obtain relevant statistical analysis. Based on our previously published and unpublished data,the suggested sample size for in vitro assays is n=6 (size effect 4.87, power: 0.99).
Data exclusions	No data were excluded
Replication	Using the data quality parameters described above, all experiments shown in the manuscript were performed independently at least twice, except the PPARG quantification by WB and the mitochondria quantification by EM.
Randomization	The allocation was random
Blinding	the in vitro experiments were performed by a single investigator therefore He was not blinded. for the in vivo experiments the investigators were blinded to the group allocation.

Reporting for specific materials, systems and methods

We require information from authors about some types of materials, experimental systems and methods used in many studies. Here, indicate whether each material, system or method listed is relevant to your study. If you are not sure if a list item applies to your research, read the appropriate section before selecting a response.

Materials & experimental systems

n/a	Involved in the study	
[ ]	[x]	Antibodies
[ ]	[x]	Eukaryotic cell lines
[x]	[ ]	Palaeontology and archaeology
[ ]	[x]	Animals and other organisms
[ ]	[x]	Human research participants
[x]	[ ]	Clinical data
[x]	[ ]	Dual use research of concern

Methods

n/a	Involved in the study	
[x]	[ ]	ChIP-seq
[ ]	[x]	Flow cytometry
[x]	[ ]	MRI-based neuroimaging

Antibodies

Antibodies used	anti-human CD3 (UCHT1, Beckman Coulter (BC) 1:50), anti-human CD4 (SFCI12T4D11, BC, 2:50), anti-human CD8 (MEM- 31, Immunotools, 1:50), anti-human CD14 (RMO52, BC, 2:50), anti-human CD15 (80H5, BC, 2:50), anti-human CD16 (3G8, BC, 1:100), anti-human CD19 (J3-119, BC, 1:100), anti-human CD20 (2H7, Biolegend, 1:100), anti-human CD33 (HIM3-4, Biolegend, 1:50), anti-human CD34 (561, Biolegend, 1:100), anti-human CD203c (E-NPP3, 1:50) (NP4D6, Biolegend, 2:50), anti-human FcεRIα (AER-37, Biolegend 2:50) anti-human CD56 (REA196, Miltenyi, 1:50). Brilliant Violet 421 anti-human CD127 (IL-7Rα) (A019D5, Biolegend, 1:100), APC anti-human CD117 (cKit) (YB5.B8, BD Bioscience, 1:50), PE anti-human CRTH2 (CD294) (BM16, Biolegend, 1:50). PE-Cy7 (JES10-5A2, Biolegend, 1:50) or PE anti-human IL-13 (7118582, BD Bioscience, 2:50) Alexa Fluor 700 anti-human AREG (In-house, 2:50) PE anti-human IL-4 (11B11, Biolegend, 1:50) and Brilliant Violet 421 anti-human IL-5 (TRFK5, Biolegend, 1:50). Brilliant Violet

605 anti-human CD3 (OKT3, Biolegend, 1:100), Alexa Fluor 700 anti-human CD4 (RPA-T4, Biolegend, 1:50), Brilliant Violet 785 anti-human CD45RO (UCHL1, Biolegend, 1:50), PE-Cy7 anti-human CXCR3 (1C6, Biolegend, 2:50), PE anti-human CRTH2 (BM16, Biolegend, 1:50), APC anti-human CD196 (CCR6) (G034E3, Biolegend, 2:50). anti-mouse CD3e (17A2, in house, 1:200), CD5 (53.7, in house, 1:200), CD19 (ID3, in house, 1:200), CD11b (M1/70, in house, 1:200), CD11c (N418, in house, 1:200), B220 (RA3-6B2, in house, 1:200), CD49b (DX5, Miltenyi Biotec, 1:200), FCεRIα (MAP-1, Miltenyi Biotec, 2:50), Ter119 (Ter119, in house, 1:200), TCRγδ (2M31/11, in house, 1:200) and TCRαβ (H57, in house, 1:200). Alexa-Fluor 700 anti-mouse CD45.2 (AL1-4A2, in house, 1:400), PE anti-mouse ST2 (RMST2-2, Invitrogen, 1:200), Brilliant Violet 605 anti-mouse CD90.2 (53-2.1, Biolegend, 1:50), PE-Cy7 anti-mouse KLRG1 (2F1/KLRG1, Biolegend, 1:200), APC anti-mouse CD117 (cKit) (2B8, eBioscience, 1:200), BV510 anti-mouse NKp46 (29A1.4 Biolegend, 1:100), BV711 anti-mouse NK1.1 (PK136, Biolegend, 1:100), BUV395 anti-mouse CD4 (GK1.5, BD, 1:200). Brilliant Violet 421 anti-mouse IL-5 (TRFK5, Biolegend, 1:50) and PEefluor610 anti-mouse IL-13 (4311635, eBioscience, 1:100).

Validation

All antibodies are commercially available and validated by manufacturer (Biolegend, eBioscience, Invitrogen, Miltenyi, BD Bioscience) additionally single staining controls were validated in the laboratory.

Eukaryotic cell lines

Policy information about cell lines

Cell line source(s)

SW1116 was purchased from ATCC; the adherent mouse MC-38 colon adenocarcinoma cell line was ordered from Kerafast, Boston.

Authentication

None of the cell lines used was authenticated.

Mycoplasma contamination

All the cell lines were regularly tested negative for Mycoplasma contamination by PCR.

Commonly misidentified lines
(See ICLAC register)

No

Animals and other organisms

Policy information about studies involving animals; ARRIVE guidelines recommended for reporting animal research

Laboratory animals

The studies involved Mus musculus laboratory females and males between 6 and 12 weeks of age bred in house. ID2-Cre ERT2 knock in mice were bred with mice containing PPARg loxP-flancked sequences. The tamoxifen-inducible cre mediated recombination resulted in the deletion of the floxed sequences in the ID2 expressing cells of the offspring. In this context Flox/flox ID2ERT2+ animals are consider as PPARg Ko and its flox/flox ID2ERT2- littermates were used as controls. RORαfl/sgIl7rCre mice were kindly provided by Prof. A McKenzie (Oliphant et al., Immunity 2014). Animals were bred and housed in the conventional UNIL-Epalinges animal facility in individually ventilated cages (Green line) under controlled light/dark and temperature cycles and ad libitum food and water. Mice were housed in the same cage at a maximum number of 5 per cage and animals were manipulated and processed following the same protocols. For in vivo tumor experiments 500,000 MC38-IL33 cells were inejected s.c in the right flank of the animals in 200ul total volume of PBS per mouse. Tumor volume was monitored and mesured using a digital caliper using the following equation: Pi/6 (D1xD2xD3) expressed as cm3.
The genotype of each animal was reveled only after the processing of the samples and FACs acquisition to avoid bias. Statistical analysis was done using GraphPad Prism software version 6. For group comparison of 2 groups a t test was used and for comparison of multiple groups a non parametric ANOVA was used. The data shown in the graphs correspond to the mean +- SEM wiht a p value < 0.05 consider statistically significant. This study was approved by the Veterinary Authority of the Swiss Canton Vaud (authorization no. 3255) and performed in accordance with Swiss ethical guidelines.

Wild animals

The study did not involve wild animals.

Field-collected samples

This study did not involve field-collected samples.

Ethics oversight

This study was approved by the Veterinary Authority of the Swiss Canton Vaud (authorization no. VD3255.f) and performed in accordance with Swiss Ethical guidelines.

Note that full information on the approval of the study protocol must also be provided in the manuscript.

Human research participants

Policy information about studies involving human research participants

Population characteristics

Healthy donors (HDs) blood was at the blood transfusion SCR centers of Lausanne, under local ethical approval.
CRC Patients: Age: 64-71; Gender: M/F; KRASmut/BRAFwt; Metastatic colorectal adenocarcinoma; Surgery and chemotherapy.

Recruitment

All patients were enrolled after the signature of an informed consent. Sample selection was done to avoid gender bias, to harmonize for age and mutation status (all patients were KRAS mut, BRAF wt).

Ethics oversight

The studies were approved by the French local and national ethics committee (CPP Grand Est II) and the Cantonal Ethics Committee of Bern. Venous blood was drawn from healthy donors (HDs) at the Swiss Transfusion Center CRSblood transfusion SCR centers of Lausanne, volunteers are asked to read an information sheet for blood donation and to complete an online medical questionnaire on the day of donation. Once the questionnaire finalized, a PDF file is generated for printing, and it is signed to give theunder local ethical approval of the blood donation.

Note that full information on the approval of the study protocol must also be provided in the manuscript.

Flow Cytometry

Plots

Confirm that:

- [x] The axis labels state the marker and fluorochrome used (e.g. CD4-FITC).
- [x] The axis scales are clearly visible. Include numbers along axes only for bottom left plot of group (a 'group' is an analysis of identical markers).
- [x] All plots are contour plots with outliers or pseudocolor plots.
- [x] A numerical value for number of cells or percentage (with statistics) is provided.

Methodology

Sample preparation	Cells were stained for 20 minutes at room temperature in 50 uL of FACS buffer. Intracellular staining was performed after fixation and permeabilization with 0.1% saponin.
Instrument	Gallios flow cytometer (Beckman Coulter), LSR-II (Becton-Dickinson, San Jose, CA), FACSAria Fusion cell sorter (BD Bioscience, San Jose, CA,USA) or the MoFlo Astrios cell sorter (Beckamn Coulter, Marseille, France).
Software	FlowJo software (TreeStar V.10)
Cell population abundance	In the post-sort fraction the abundance of the relevant cell population was equal or higher than 95%. the purity was determined by requiring an aliquot of the sorted sample.
Gating strategy	Human ILCs are identified by combining fluorescently labelled antibodies specific for Lineage markers, CD127, c-Kit (CD117), CRTH2 and a dead/live cell discrimination marker. By this strategy, populations of interest are defined as: ILC1s (Lineage-, CD127+, CRTH2-, c-Kit-); ILC2s (Lineage-, CD127+, CRTH2+, c-Kit+/-) and ILCPs (Lineage-, CD127+, CRTH2-, c-Kit+).<br>Mouse ILCs were sorted as CD45+, Lin-, CD90+ lymphocytes. ILC1s were sorted as NK.1.1+,NKp46+; ILC2s as ST2+, KLRG1 +/-; ILC3s as c-KIT+

- [x] Tick this box to confirm that a figure exemplifying the gating strategy is provided in the Supplementary Information.
